# Wireless Ultrasonic Sensing for Fatigue Crack Propagation and Life Prediction in Thin Plate Structures

**DOI:** 10.3390/s26113357

**Published:** 2026-05-26

**Authors:** Shuo Chen, Jiahang Du, Minsheng Liu, Qiuyu Peng, Jiayi Mi

**Affiliations:** 1 State Key Laboratory of Precision Blasting, Hohai University, Nanjing 210024, China; 2 College of Civil and Transportation Engineering, Hohai University, Nanjing 210098, China; 231304020005@hhu.edu.cn (J.D.); lms961015@163.com (M.L.); 241304020022@hhu.edu.cn (Q.P.); mi15846779315@163.com (J.M.); 3 Research and Development Department, Hangzhou Kuangxing Technology Co., Ltd., Hangzhou 311107, China

**Keywords:** wireless sensing, ultrasonic nondestructive evaluation, fatigue test, thin plate structure, fatigue crack evaluation

## Abstract

Recent advancements in sensor technology have made in-situ crack assessment of structures feasible. To investigate the correlation between the ultrasonic amplitude and metal fatigue life, an aluminum compact tension (C(T)) specimen was fabricated to simulate fatigue damage in thin plate structures. An experimental investigation of fatigue crack propagation was performed, wherein the specimen experienced cyclic uniaxial tensile loading at constant amplitude. The crack propagation behavior was analyzed, and the relationship between crack length and the associated loading cycles was determined. Additionally, the evolution of the ultrasonic signal during crack propagation was investigated, and the quantitative dependence of the ultrasonic characteristic parameter on crack length was revealed. Finally, a model correlating ultrasonic characteristic parameters with loading cycles was developed, enabling fatigue life evaluation. The proposed method demonstrates significant potential for evaluating the fatigue life of thin plate structures.

## 1. Introduction

Thin plate structures are extensively employed in wind turbine blades, ship decks, and steel bridges, where they inevitably experience cyclic loading during their operational lifespan. Steel bridges are estimated to experience approximately 1000 repetitive loads from fully loaded trucks per day, resulting in the accumulation of more than 2.7 × 10^7^ loads over a 75-year service life [1]. Therefore, accurate assessment and timely repair of fatigue damage are essential for prolonging structural service life, making fatigue crack monitoring critically important. Experimental testing represents one of the most direct and reliable approaches, and numerous methods have been developed for this purpose [2,3,4,5,6].

Albert published the first fatigue test results in 1837 [7], and research on metal fatigue has spanned more than a century and yielded remarkable progress. Wöhler employed a rotary bending test machine to conduct systematic fatigue tests [8], reported that the stress amplitude influences the crack growth rate, and proposed a stress–life (*S*-*N*) curve. In 1963, Paris developed an empirical relation linking the crack propagation rate to the variation in stress intensity factor (SIF) [9], forming the foundation for predicting fatigue life. The notion of the effective SIF range was introduced by Elber, who identified crack closure and used it to extend Paris’s law [10]. Elber’s study demonstrated the necessity of analyzing elastoplastic materials using elastoplastic fracture mechanics methods. Subsequent studies [11,12,13,14,15,16] have revealed that the effective SIF range ratio is affected by multiple factors, including the loading ratio, specimen type, geometry, material, and loading history. The studies [17,18] collectively provide a critical theoretical basis for quantitatively characterizing and estimating fatigue crack propagation under diverse loading scenarios and specimen geometries.

For the purpose of metallic fatigue crack detection, several techniques, such as wired strain gauge monitoring [19], eddy current testing [20], and vision-based detection [21], are widely utilized and have been proven to be effective. However, due to limitations such as complex installation requirements, labor-intensive procedures, and high energy consumption, these methods are difficult to deploy and maintain on large-scale or complex structures. Ultrasonic inspection, on the other hand, is a widely employed technique for assessing fatigue cracks in thin plate structures owing to its high resolution, sensitivity, accuracy in crack localization, and adaptability to material properties [22]. Considerable research has been carried out to clarify the relationship between ultrasonic signal parameters and crack size through both experimental and theoretical approaches [23,24,25,26]. Owing to their favorable signal-to-noise ratio, fine resolution, and strong interference resistance, Rayleigh waves have become important tools in nondestructive testing (NDT) [27]. Given the stringent requirements for accuracy and efficiency in detecting fatigue damage in thin plates, Rayleigh waves were adopted as the detection method in this study.

With the increasing demand for field monitoring of cracked structural components, wireless ultrasonic sensing devices have been developed, offering on-board data processing, low cost, energy efficiency, and miniaturization. However, these technologies have not been fully applied in engineering practice, partly because a robust quantitative model for in-situ monitoring using wireless systems has not been fully established. This study explores the potential of using ultrasonic signals to assess fatigue cracks. Fatigue experiments were conducted on thin plate specimens fabricated from 6061 aluminum alloy. Ultrasonic evaluations were performed through a self-developed wireless ultrasonic sensing system [28]. This study analyzed crack extension and investigated its correlation with changes in ultrasonic signal characteristics. Considering the correlation between crack length and ultrasonic signal characteristics along with the effect of crack closure, a model describing crack propagation under fatigue was established. The proposed model is strongly consistent with the experimental results. Beyond the technical advantages of being compact in size and accurate in detection, the wireless sensing device and coordinated quantitative evaluation method in this paper offer the potential for continuous, in-situ infrastructure monitoring. This practical capability could benefit the optimization of maintenance schedules and more efficient resource allocation for targeted repairs.

## 2. Experimental Setups

### 2.1. Analysis of the Influence of Probe Placement on Test Results

During the testing of C(T) specimens, considering the asymmetry introduced by the notch in the ultrasonic propagation path and the disturbance of probe positioning caused by equipment vibration during loading is important. These factors can cause misalignment between the transmitting and receiving ultrasonic probes, displacing them from a common centerline. Therefore, investigating how variations in probe placement affect the amplitude of the received signal envelope is necessary.

**Figure 1:**
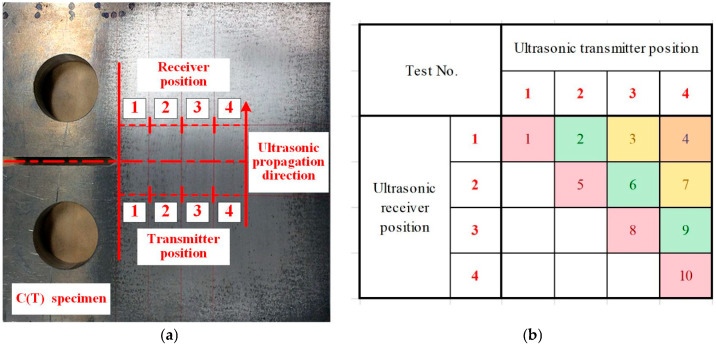
Schematic diagram of the impact test probe layout and experimental design. (**a**) Schematic diagram of sensor placement; (**b**) correspondence between the serial number of the experimental group and the position of the probe.

The specimens were prepared using 6061 aluminum alloy. As shown in Figure 1a, the transmitting unit and the receiving unit of the ultrasonic system were placed at a central spacing of 25.4 mm. A total of four transmitting and receiving ultrasonic probes were arranged, with a spacing of 12.7 mm between them, and ultrasonic signals were acquired at a 2 MHz sampling frequency. To evaluate the influence of probe placement on the amplitude of the received signal, ten sets of ultrasonic signal cross-acquisitions were conducted at four positions, as shown in Figure 1b.

**Figure 2:**
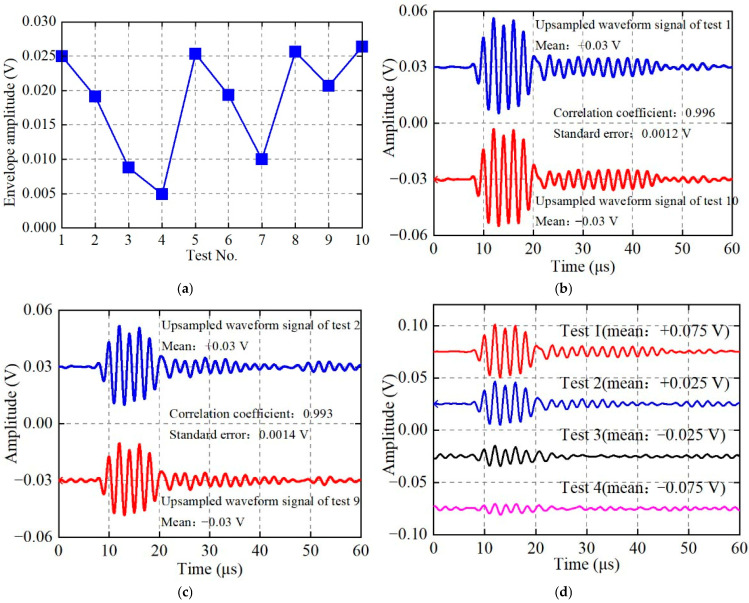
Diagram of signal amplitude and partial waveform variation with probe position. (**a**) Variation in signal amplitude and waveform with probe position; (**b**) comparison of acquired waveforms between test 1 and test 10; (**c**) comparison of acquired waveforms between test 2 and test 9; (**d**) comparison of acquired waveforms from tests 1, 2, 3, and 4.

The envelope of ultrasonic signals processed at a sampling frequency of 250 MHz is shown in Figure 2a. When the transmitting and receiving probes were aligned at the same relative positions, for example, in test group No. 1, 5, 8, and 10, test group No. 2, 6, and 9, and test group No. 3 and 7, the amplitudes of the received signals were nearly identical. Moreover, the amplitudes increased as the distance from the notch and opening increased. For example, the signal amplitude in test 10 was 5.42% greater than that in test 1, whereas that in test 9 was 8.15% greater than that in test 2. These results indicate that the interference from the notch and opening gradually decreases as the probe distance increases. Furthermore, the results confirm that ultrasonic waves not only propagate along their primary paths but also exhibit some dispersion into surrounding regions, although most of the excitation energy remains concentrated along the main paths. For example, the signal amplitude in test 4 decreased by 80.43% compared with that in test 1. When the transmitting probe remained fixed, the amplitude of the collected signal decreased as the receiving probe gradually shifted. Therefore, maintaining proper alignment between transmitting and receiving probes is essential during signal acquisition. The influence of the waveform characteristics of the acquired signals on the results is shown in Figure 2b and Figure 2c, respectively. A comparison of waveform signals from tests 1 and 10 and tests 2 and 9 revealed that the notch and opening had little effect on the waveform shape. Therefore, compensation of the acquired waveform signals at different positions was not required in the subsequent fatigue crack analysis.

**Figure 3:**
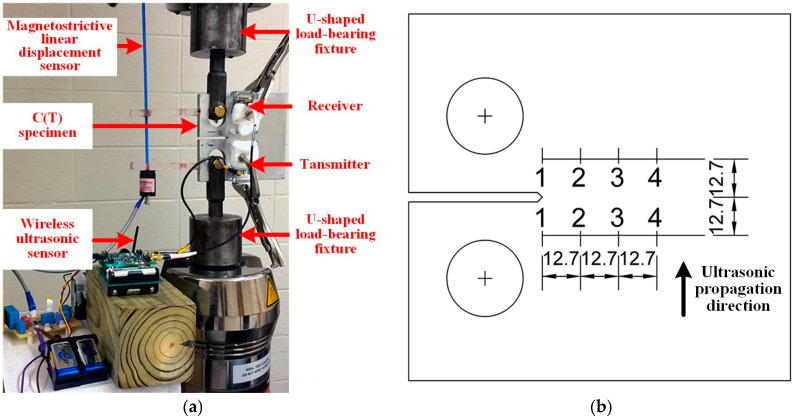
Schematic diagram of wireless signal collection experiment design for C(T) specimen fatigue cracks. (**a**) Experimental layout diagram; (**b**) dimensional drawing of the probe position arrangement.

The waveforms acquired in tests 1–4, which were designed to study the effect of moving the receiving probe while keeping the transmitting probe fixed, are shown in Figure 3a. The first set of peaks appeared in the received signals at 10–20 μs. As the probe spacing increased, the waveform shape remained unchanged, but the amplitude decreased sharply. This behavior corresponds with the observations of Ohara et al. [29], who described favorable wavefront spacing during ultrasonic imaging of surface cracks in C(T) specimens using subharmonic phased array technology. The second group of peaks appeared at 20–45 μs. With increasing probe spacing, the waveform shape again remained unchanged, but the amplitude continued to decrease, and the duration was significantly shorter than that of the first group of peaks. This finding indicates that the reflected wave also exhibited diffusion while maintaining excellent directionality. These findings are consistent with the waveform changes in ultrasonic reflections from isotropic solids reported by Rose et al. [30].

### 2.2. Experimental Preparation and Scheme Design

The specimen dimensions are shown in Figure 3b, and both the specimen and U-grip comply with ASTM standards. To reduce the impact of surface roughness on ultrasonic wave transmission, the specimen surface was polished with sandpaper before experimentation. The specimen was mounted in the fixture by adjusting the screws, ensuring that the upper and lower grips were aligned along the same vertical axis.

**Figure 4:**
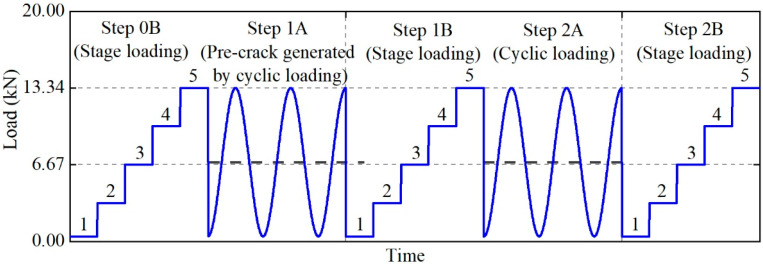
C(T) specimen fatigue test loading time–history curve.

The variation in the load with time during the specimen loading process is shown in Figure 4. Owing to restrictions in the loading apparatus, applying a minimum load of 0 kN could lead to unintended compressive stresses. Therefore, the minimum load was set to 0.44 kN to avoid this issue. During the pre-crack and propagation stages, the applied load ranged from 0.44 kN to 13.34 kN, resulting in an actual load ratio of 0.033. The loading procedure is illustrated in Figure 4, and the detailed parameters are given in Table 1. The loading mode between 0.44 kN and 13.34 kN was multistage static loading, which was divided into five levels. Notably, the stress state of the specimen may influence the amplitude of the ultrasonic signal. Therefore, a set of reference signals (at step 0B) was collected before the formal test.

## 3. Results and Discussion

Measurements were obtained through fatigue crack propagation and ultrasonic testing experiments. First, the crack size measurements were verified for compliance with ASTM E647-24 requirements [31]. Next, the data were screened according to the relative position of the ultrasonic probe to the crack. A detailed discussion of fatigue crack behavior is presented, focusing on the correlation of crack length with loading cycles. In addition, the variations in ultrasonic signal waveforms and envelope amplitudes during fatigue crack propagation are analyzed and discussed.

### 3.1. Validation of Fatigue Test Data and Fracture Morphology Analysis

**Figure 5:**
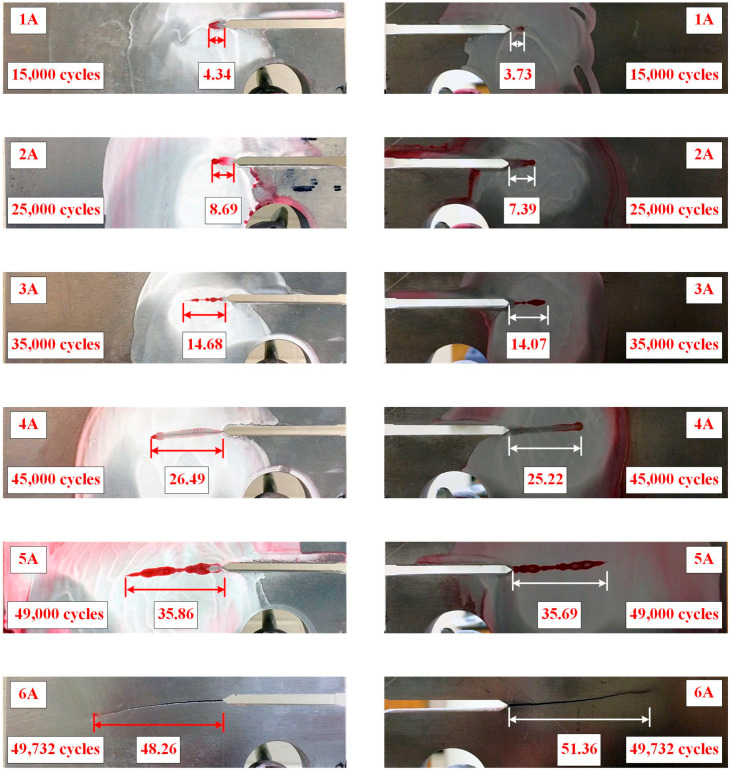
Crack morphology diagrams of the C(T) specimen on both the front and back sides under different fatigue cycles (unit: mm).

The morphology and length of cracks after various loading cycles under a tensile load of 13.34 kN are presented in Figure 5. In accordance with ASTM standards, four main criteria are used to validate crack data: crack length, difference between the front and back crack lengths, discrepancy between the mean left and right crack lengths, and deviation angle.

As shown in Figure 5(1A), the shortest prefabricated crack measured 3.73 mm, which exceeded 0.1 times the specimen thickness (1.27 mm), notch width (3.18 mm), and 1 mm threshold. A discrepancy of 0.61 mm was found between the front and back crack lengths, which is less than the threshold of 0.25 times the specimen thickness. The maximum deviation angle of the fatigue crack was 5.1°, which is less than the 10° limit specified by the standards. Therefore, all fatigue crack dimensions comply with the specifications, confirming the validity of the experimental data. These results confirm that the C(T) specimen design and loading procedure are appropriate and that the measured crack length data are suitable for a subsequent analysis of fatigue crack propagation.

### 3.2. Fractographic Analysis

**Figure 6:**
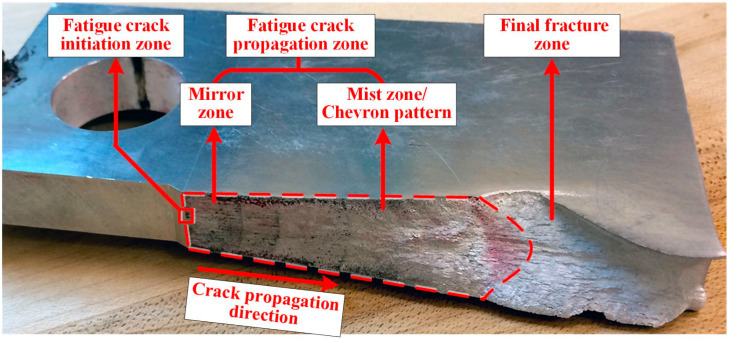
Fracture surface morphology diagram of the C(T) specimen.

Fractographic analysis is an effective method for understanding the mechanisms of specimen failure during the loading process [32]. As shown in Figure 6, surface smoothness decreases progressively along the fatigue crack propagation path. Morphological analysis revealed three characteristic regions on the fatigue fracture surface: crack initiation, crack propagation, and final fracture. The crack initiation zone is characterized by discrete areas located at the notch root in proximity to both specimen surfaces. The measured crack extension along the front surface was greater than that on the rear surface, which to some extent confirms the validity of the defect location.

Along the propagation direction, within the crack propagation region, three distinct subregions can be identified: mirror, mist, and hackle. The transition between the mist and hackle regions was gradual, and their boundary was not distinct. The fracture in the final fracture region was perpendicular to the axial tensile load, which is consistent with the characteristics of a normal fracture. Additionally, a shear lip region characterized by plastic deformation at an angle of approximately 45° formed between the fracture plane and the 0.5 mm wide upper and lower specimen surfaces.

Numerous concentric arc lines perpendicular to the local crack direction appeared on the fracture surface in the final fracture region, which is consistent with typical fatigue features. This finding also indicates that the specimen experienced significant plastic deformation during further crack propagation. With increasing loading cycles, the crack continued to propagate, and the effective specimen area decreased progressively. When the effective specimen area was no longer sufficient to resist the external load, fracture occurred in the final fracture region. Fractographic observations of the final fracture region revealed that the angle between the failure surface and the tensile axis was approximately 45°, indicating that the final failure was induced by shear strain.

### 3.3. Crack Growth Rate Analysis

Investigating crack propagation under repeated loading cycles plays a crucial role in characterizing fatigue behavior and estimating service life. Owing to substantial differences in crack length among specimens subjected to identical loading cycles, the behavior of crack growth is typically quantified using the crack growth rate, *da*/*dN*, where *a* denotes the crack length and *N* represents the number of loading cycles. After various models used to describe crack growth behavior [33] were compared, this study adopted the modified secant method and the five-point incremental polynomial method. According to Equations (1) and (2), the crack propagation rate and the corresponding SIF values were determined.

(1) $${\left(da/dN\right)}_{i}=\frac{1}{2}\left(\frac{a_{i+1}-a_{i}}{N_{i+1}-N_{i}}+\frac{a_{i}-a_{i-1}}{N_{i}-N_{i-1}}\right)$$

(2) $$\left\{\begin{array}{l} \Delta K_{i}=\frac{\Delta P}{B\sqrt{W}}Y_{i}\\ Y_{i}=\frac{2+\alpha_{i}}{{\left(1-\alpha_{i}\right)}^{3/2}}\left[0.886+4.64\alpha_{i}-13.32\alpha_{i}^{2}+14.72\alpha_{i}^{3}-5.6\alpha_{i}^{4}\right]\\ \alpha_{i}=\frac{\alpha_{i}}{W} \end{array}\right.$$

where (*da*/*dN*)*_i_* represents the fatigue crack growth rate, *a_i_* is the average crack length, and Δ*K_i_* denotes the SIF range. Here, Δ*P* denotes the loading range, *W* refers to the specimen width, and *B* refers to its thickness.

Based on the established literature, the five-point incremental polynomial method outperforms the seven-point method in terms of accuracy, particularly when the data are limited [34]. For this reason, the five-point method was employed in this work. The fitted crack length $\hat{a}$*_i_* is then determined as follows:

(3) $$\left\{\begin{array}{l} {\hat{a}}_{i}=b_{0}+b_{1}H_{i}+b_{2}H_{i}^{2}\\ H_{i}=\left(N_{i}-C_{1}\right)/C_{2}\\ C_{1}=\left(N_{i+2}+N_{i-2}\right)/2\\ C_{2}=\left(N_{i+2}-N_{i-2}\right)/2 \end{array}\right.$$

In Equation (3), the variables *b*_0_, *b*_1_, and *b*_2_ represent the regression parameters, which are obtained using the least-squares fitting method. The input parameters *C*_1_ and *C*_2_ are scaling factors used to normalize the data. The expression for the crack growth rate is obtained by differentiating Equation (3) with respect to *N*, as shown below:

(4) $${\left(da/dN\right)}_{i}=b_{i}/C_{2}+2b_{2}\left(N_{i}-C_{1}\right)/C_{2}$$

Furthermore, the SIF range Δ*K_i_* associated with (*da*/*dN*)*_i_* was calculated according to Equation (2). In particular, the fitted crack length $\hat{a}$*_i_* replaces the measured crack length in calculations to minimize the effect of experimental errors.

**Figure 7:**
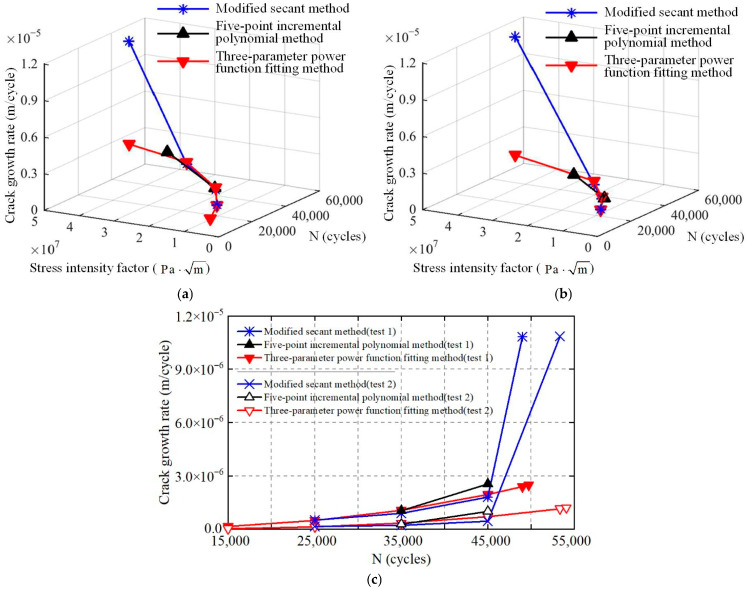
Crack growth rate variation curves for two sets of parallel experiments fitted by three different methods: (**a**) Experiment 1 (Section 3.1); (**b**) Experiment 2 (parallel test); (**c**) comparison of *da*/*dN* versus *N* curves from three methods.

To improve data accuracy, the SIF range and the crack growth rate were obtained through the experimental results using the modified secant method and the five-point incremental polynomial method, respectively. The relationships among the fatigue life, crack growth rate, and range of the SIF are shown in Figure 7a and Figure 7b, respectively. From a macroscopic perspective, considering the order of magnitude and trend of the fitted results, the spatial curves obtained from two sets of experiments using the same fitting method show a high degree of consistency. These findings indicate that both methods are highly stable for fitting the data of C(T) specimens in constant-amplitude crack growth tests.

However, Figure 7c illustrates that the crack growth rate determined via the five-point incremental polynomial method surpasses that derived from the modified secant method [33]. During the near-failure stage, when the number of cycles exceeds 45,000, the crack growth rate calculated through the modified secant method increases rapidly and aligns more closely with the experimental trends.

The SIF range represents the stress state and the crack propagation capacity near the crack tip [35]; therefore, its continuous increase with the number of loading cycles leads to an expansion of the plastic zone surrounding the crack. This dynamic results in a greater number of active cracks and an increased crack propagation rate. As the specimen approaches failure, a pronounced increase occurs simultaneously in the SIF range and crack growth rate.

### 3.4. Discussion of the Ultrasonic Measurement Results

**Figure 8:**
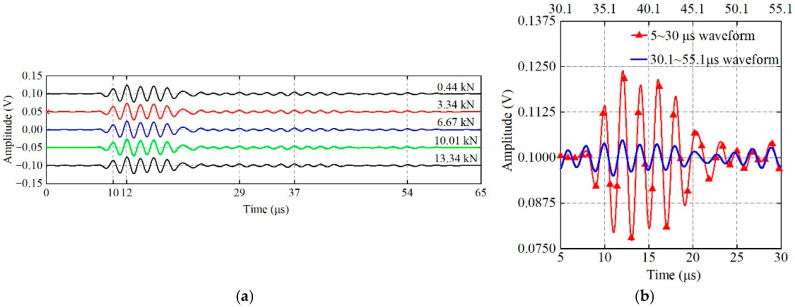
Waveforms of the wireless ultrasonic signals. (**a**) Waveform variation at step 1B, position 3 under different tensile loads; (**b**) waveform superposition at 0.44 kN tensile load.

Based on the experimental schedule presented in Table 1, ultrasonic signals acquired by the wireless sensor network were up-sampled from 2 MHz to 250 MHz with baseband sine functions, and the peak amplitudes were then computed by applying the discrete Hilbert transform. Figure 8a,b show two representative sets of signal waveforms. To facilitate comparison, the signals were adjusted by time-shifting and mean-centering so that their peaks coincided at 12 μs. In addition, all the waveforms were baseline-shifted to 0 V to provide equal spans.

The variations in the wireless up-sampled ultrasonic waveforms under different tensile loads at step 1 and position 3 are shown in Figure 8a. The waveforms exhibit high consistency, with correlation coefficients between any two signals ranging from 0.9985 to 0.9999. The amplitude differences range from 5.92 × 10^−5^ V to 3.90 × 10^−4^ V. No clear relationship is observed between the correlation coefficients and amplitude differences. However, all the coefficients exceed 0.998, indicating a high degree of waveform consistency, while the standard error remains below 3.90 × 10^−4^ V, suggesting a negligible impact.

Furthermore, four distinct peaks appear within the first 65 μs, occurring at 12 μs, 28.9 μs, 37.1 μs, and 54 μs. Among them, the peak at 12 μs is the largest in the sequence, the peaks at 28.9 μs and 37.1 μs are approximately equal, and the peak at 54 μs is the smallest. Considering the consistent arrival time difference of 16.9 μs between the first two and the last two peaks and the fact that the main peaks follow a “larger–smaller” pattern, two waveform segments were extracted and overlapped for comparative analysis, as shown in Figure 8b.

**Figure 9:**
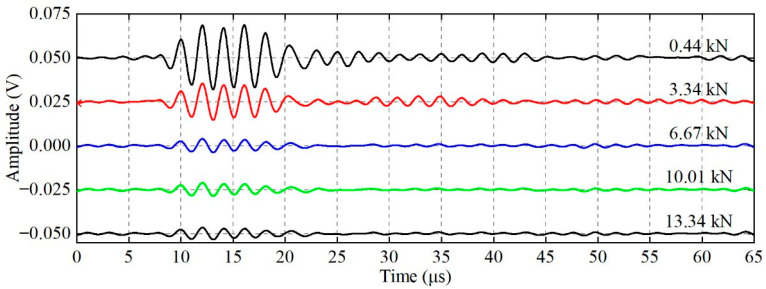
Comparison chart of wireless up-sampled ultrasonic signal waveforms under different tensile loads at step 4B and position 1.

The data in Figure 9 illustrate that not only do the main peaks of the two waveforms coincide in position but the secondary peaks also occur at identical positions, indicating consistent relative trends. This finding indicates that the second set of waveforms is closely associated with the first set during the formation process. Considering the geometric configuration of the C(T) specimen and the findings of Golan et al. [36], the second set can be identified as the reflected wave of the first, with an arrival time delay of 25.1 μs. According to the waveform analysis in Section 2.1, the signal at approximately 30 μs originates from reflection at the back surface. Accordingly, the peaks at 12 μs, 28.9 μs, 37.1 μs, and 54 μs can be attributed to surface-to-surface transmission, reflection from the back surface, surface wave reflection at the crack tip, and reflection from the crack tip induced by the back surface reflected wave, respectively.

### 3.5. Determination of the Fatigue Crack Opening Force

The fatigue crack opening force must be determined to evaluate how crack closure influences fatigue crack propagation; this further provides the foundation for adjusting the crack growth rate curve to incorporate the closure effect. In this section, following the ASTM E647 standard [31] and applying the crack mouth opening displacement (CMOD) method, the fatigue crack opening force is indirectly obtained using magnetostrictive linear displacement sensor data through compliance curve calculations.

**Figure 10:**
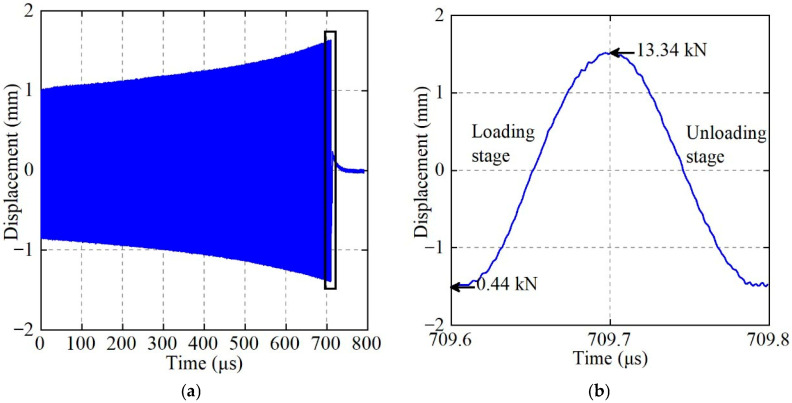
Crack mouth opening displacement versus time curve as step 5A approaches the end of loading. (**a**) Macroscopic curve of displacement versus time; (**b**) locally enlarged section of displacement–time curve (boxed region).

The displacement variation curve within the last 800 s of cyclic loading recorded by the magnetostrictive linear displacement sensor at a sampling frequency of 1000 Hz under step 5A is shown in Figure 10a. The displacement corresponding to an average load of 6.89 kN is defined as 0 mm. As shown in the figure, with increasing loading cycles and crack length, the CMOD increases correspondingly. Because the CMOD reflects the resistance of the crack tip to propagation, this resistance decreases as the specimen approaches failure and as the number of load cycles increases. The displacement–time curve of the final loading cycle in step 5A, corresponding to the shaded region in Figure 10a, is shown in Figure 10b. The $\delta_{amp}$ is defined by Equation (5):

(5) $$\delta_{\mathrm{amp}}=\delta -\delta_{\min}$$

In Equation (5), $\delta_{amp}\;$ denotes the CMOD (unit: mm), δ represents the crack displacement (unit: mm), and $\delta_{min}$ denotes the displacement associated with the minimum load of the loading cycle (0.44 kN in this section), which is also in millimeters.

**Figure 11:**
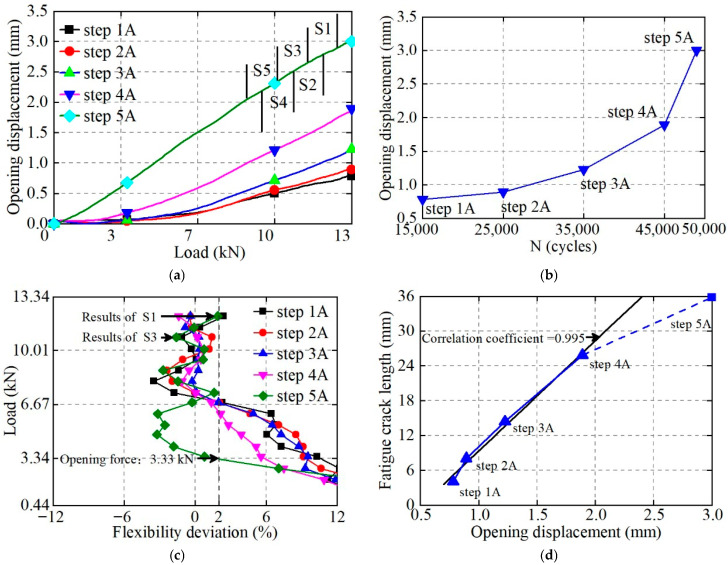
Variation curves of crack mouth opening displacement and its computational parameters at different loading stages. (**a**) Flexibility curve of loading segment at the last cycle load of different steps; (**b**) curve of crack mouth opening displacement versus cycle number; (**c**) curve of flexibility deviation versus load; (**d**) curve of maximum opening displacement versus fatigue crack length.

The projected fatigue crack length at the midpoint of the inclined wedge after 45,000 loading cycles measuring 25.86 mm is shown in Figure 11. The wireless up-sampled ultrasonic waveforms collected at position 1 under varying tensile loads exhibit marked variation. In particular, the peak amplitude at 12 μs clearly decreases. Additionally, the arrival times of signals occurring after 25 μs are progressively delayed with increasing sustained tensile load. During fatigue testing, the crack penetration state, its relative position with respect to the inclined wedge, and its load-induced transition from closure to opening remained consistent. Therefore, the observed changes result from the combined effects of the evolving specimen stress state and the transition of the crack from closed to open under tensile loading [37].

According to Equation (5), the CMOD for the final loading segment of each cyclic step is calculated, and the corresponding compliance curve is plotted, as depicted in Figure 11a. The maximum CMOD to the maximum load at different cycle counts is extracted from Figure 11a and plotted against the cumulative number of cycles, yielding the curve in Figure 11b. As shown in Figure 11b, as the number of cycles increases, the growth rate of the maximum CMOD gradually accelerates. The underlying mechanism is further examined using the crack opening force determined at different loading stages, as presented in Figure 11c.

As illustrated in Figure 11c, the compliance deviation curves versus load were obtained following the procedure according to the ASTM standard E647-24 [31]. According to this procedure, the load at which the compliance deviation reaches 2% is regarded as the crack opening force for each loading step. Here, the compliance deviation $\delta_{offset}$ is defined by Equation (6):

(6) $$\delta_{\mathrm{offiset}}=\frac{\delta_{\mathrm{open}-\mathrm{crack}}-\delta_{\mathrm{compliance}}}{\delta_{\mathrm{compliance}}}\times 100\%$$

In Equation (6), $\delta_{offset}$ represents the percentage of compliance deviation; $\delta_{open-crack}$ denotes the opening crack compliance, defined as the gradient of the line obtained by linearly fitting the unloading segment of the compliance curve in the 70–95% load range; and $\delta_{\mathrm{compliance}}$ represents the segment compliance, which is calculated by fitting straight lines over a 10% load range with a 5% overlapping segment starting from 95% of the load. The slope of each segment is taken as its compliance, and the average load corresponding to that segment is taken as its representative load. The variation in the CMOD with fatigue crack length is shown in Figure 11d. Approximating the crack geometry as an isosceles triangle allows for the ratio of CMOD to the fatigue crack length to quantify the crack tip opening displacement. Consequently, the slope of the curve in Figure 11d reflects this displacement across various loading steps. Linear regression was performed on the first four data points using the least squares method. The resulting correlation coefficient of 0.995 indicates excellent linearity, suggesting that the crack-tip opening displacement remains nearly constant. These observations agree with the findings of Solanki [38], who developed a two-dimensional model for C(T) specimens. Their numerical simulations revealed that during the phase of steady crack growth, the plastic zone around the crack tip remains consistent, and the opening displacement at the crack tip remains constant under identical loading conditions.

### 3.6. Pattern of Amplitude Variation in Signals Collected Wirelessly

**Figure 12:**
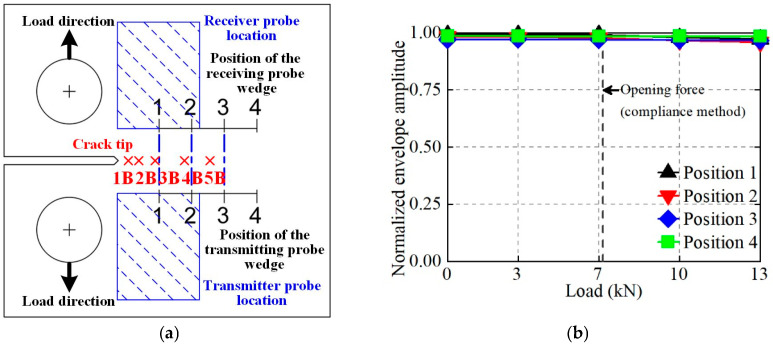

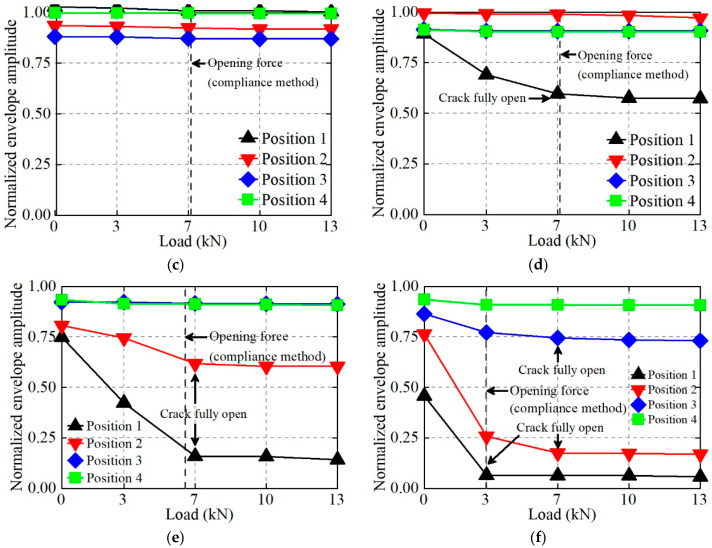
Schematic diagram of relative probe-crack position and the corresponding variation curve of the normalized envelope amplitude of the ultrasonic signal. (**a**) Schematic of relative position between crack tip and probe; (**b**) normalized envelope amplitude of ultrasonic signal at step 1B; (**c**) normalized envelope amplitude of ultrasonic signal at step 2B; (**d**) normalized envelope amplitude of ultrasonic signal at step 3B; (**e**) normalized envelope amplitude of ultrasonic signal at step 4B; (**f**) normalized envelope amplitude of ultrasonic signal at step 5B.

The spatial relationship between the fatigue crack tip and the centerline of the inclined wedge of the probe at various experimental stages is shown in Figure 12a. The variation curves of the normalized envelope amplitude of the wireless ultrasonic transmission signals are shown in Figure 12b,f. These signals were collected under different fatigue cycles, positions, and tensile loads and are plotted against the crack length. The normalized envelope amplitude is calculated according to Equation (7) as follows:

(7) $$T=\frac{A^{\mathrm{env}}}{A_{1B}^{\mathrm{env}}(1)}$$

In this equation, *T* represents the transmission coefficient. The term *A*^env^ is defined as the envelope amplitude acquired at a designated holding load following a specified quantity of loading cycles, while $A_{1B}^{env}(1)$ serves as the baseline envelope amplitude, collected under a 0.44 kN holding load after the initial pre-crack.

Based on the relative probe-crack position depicted in Figure 12a and the corresponding observations in Figure 12b,c, the projected inter-wedge crack lengths for steps 1B and 2B are determined to be 4.40 mm and 8.06 mm, respectively. The envelope amplitude of the ultrasonic signals measured by the probe under different tensile loads does not significantly fluctuate. In Figure 12d, during signal collection at step 3B, the projected crack length between the wedges is 14.38 mm. At this stage, the envelope amplitude measured by the probe at position 1 fluctuates noticeably with the load, whereas the curves from the other probe positions show no significant fluctuation. Similarly, in Figure 12e,f, it is evident that the envelope amplitude measured by a probe fluctuates noticeably with the load once the crack tip moves beyond the corresponding location. In contrast, probes located at positions not yet crossed by the crack tip show no significant fluctuation. Therefore, the ultrasonic probe can effectively detect fatigue cracks only when they are located within the wedge range and have a projected length greater than 10.39 mm.

## 4. Model for Fatigue Loading Cycles and Envelope Amplitudes of the Ultrasonic Signals

In this section, a model is formulated to determine the correlation between crack length and loading cycles, taking the crack closure effect into account. This is achieved by combining crack propagation analysis with Paris’s and Miner’s laws. The determination of the crack tension load is based on the analysis of ultrasonic waveform changes. However, identifying the model parameters is crucial yet complex. Previous studies [39] have shown that these parameters and ultrasonic signal variations strongly depend on the specimen material. On this basis, we examined the correlations among the crack length, loading cycle count, and ultrasonic transmission coefficient. Ultimately, we formulated a model that links loading cycles with transmission coefficients via crack length, which is in good agreement with the experimental data.

### 4.1. Model for the Crack Length and Number of Fatigue Loading Cycles

Paris’s law, which is typically used to describe crack growth under fatigue loading, is expressed as follows:

(8) $$\log\left(da/dN\right)=m\log(\Delta K)+\log C$$

In this expression, *da*/*dN* signifies the crack growth rate, while Δ*K* is the SIF range. The coefficients *C* and *m* are properties that are dependent on the material. To include the impact of crack closure, the standard SIF range (Δ*K*) is substituted with the effective SIF range, denoted as Δ*K_eff,i_*, with its definition given below:

(9) $$\left\{\begin{array}{l} \Delta K_{eff,i}=\frac{\Delta P_{eff.i}}{B\sqrt{W}}Y_{i}\\ Y_{i}=\frac{2+\alpha_{i}}{{\left(1-\alpha_{i}\right)}^{3/2}}\left[0.886+4.64\alpha_{i}-13.32\alpha_{i}^{2}+14.72\alpha_{i}^{3}-5.6\alpha_{i}^{4}\right]\\ \alpha_{i}=\frac{a_{i}}{W} \end{array}\right.$$

Here, *B* and *W* represent the thickness (0.0127 m) and width (0.1016 m) of the specimen, respectively. The term Δ*K*_eff*,i*_ is the effective SIF range after *N_i_* cycles, corresponding to the average fitted crack length *a_i_* at that point. The effective amplitude of tensile loading subsequent to *N_i_* cycles, denoted as Δ*P*_eff*,i*_, is determined by the following expression:

(10) $$\Delta P_{\mathrm{eff},i}=P_{\max}-P_{\mathrm{op},i}$$

In this study, the maximum tensile load, *P*_max_, is 13,340 N. The fatigue crack opening force, *P*_op,*i*_, is simplified based on the analysis in Section 3.4. For simplicity, the fatigue crack opening force for C(T) specimens can be likened to the response of dam gates to water levels. If the mean fatigue crack length is less than 2.586 × 10^−2^ m, the gates remain fully open with a force of 6670 N. When the crack length exceeds this threshold, the gates partially close, reducing the force to 3340 N.

Based on Equation (8), *da*/*dN* and the corresponding Δ*K_eff,i_* values were recomputed using both the modified secant method and the five-point incremental polynomial approach. By substituting Δ*K* with Δ*K_eff,i_*, regression analysis yields the parameters log*C* and *m*. A comparison of the results before and after this modification is presented in Table 2. log*C*_eff_ = log*C* is defined. In addition, *m* and Δ*K* are replaced by *m*_eff_ and Δ*K_eff_*, respectively, and Equation (8) can be reformulated as follows:

(11) $$dN=C_{eff}^{-1}{\left(\Delta K_{eff}\right)}^{-m_{eff}}da$$

Based on the method introduced in Section 3.2, *C*_eff_ and *m*_eff_ were calculated. Miner’s rule states that given an initial average crack length *a*_0_, the corresponding increase in crack length can be represented as $\Delta a_{0}=a-a_{0}$, and the corresponding definite integral expression for the estimated increase in loading cycles is given by the following equation:

(12) $$\Delta N={\int_{a_{0}}^{a_{0}+\Delta a}C_{C(T)}^{-1}\left(\Delta K_{eff,i}\right)}^{-m_{eff}}da$$

**Figure 13:**
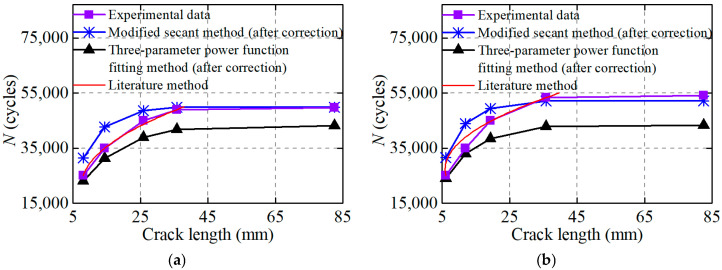
The predicted curve of fatigue crack length versus number of cycles after modification: (**a**) Experiment 1 (Section 3); (**b**) Experiment 2 (parallel experiment).

In this expression, Δ*K_eff_* denotes the effective SIF range corresponding to a fatigue crack length varying from a0 to ac. By using the *da*/*dN* data and the corresponding Δ*K_eff_* values, the loading cycles are derived according to Equation (10). The relationships among the measured data, calculated data, and crack length are illustrated in Figure 13.

The predicted curves from both experimental sets are consistent with the experimental results (Figure 13). In addition, the curves are also compared with those calculated using the method from the literature [40]; all of the methods demonstrate good agreement in the trends. The established model from the literature shows strong agreement during the stable crack propagation stage. However, it results in a higher prediction error of ultimate fatigue life. After calculation, the correlation coefficients between the predicted and experimental curves before and after applying the modified secant method are 0.979, 0.978 and 0.962, 0.961 for the two experimental sets, respectively. The standard errors decrease from 11,568 cycles and 12,347 cycles before correction to 4801 cycles and 5438 cycles after correction, corresponding to reductions of 42% and 44%, respectively, which demonstrates a marked improvement. Compared with the uncorrected curves, the corrected curves fit the experimental curves more closely. Compared with the experimental results, the predicted fatigue life changes from 29% and 31% underestimated before correction to 0.4% overestimated and 4% underestimated after correction, indicating a significant improvement. Similar conclusions are obtained from the predicted curves generated by the five-point incremental polynomial method. The correlation coefficients between the predicted and experimental curves before and after correction are 0.997, 0.999 and 0.993, 0.993, respectively, for the two sets of experiments, whereas the curve shapes remain largely unchanged. The standard errors decrease from 19,966 cycles and 21,408 cycles before correction to 5478 cycles and 7393 cycles after correction, corresponding to reductions of 27% and 35%, respectively, which again demonstrates a significant improvement. These results indicate that the established *a*-*N* relationship accurately characterizes the crack propagation process of C(T) specimens. It is also notable that a two-set parallel test was conducted to assess the stability of the proposed method, and the results demonstrated a good agreement in both the numerical values and the overall trend. However, comprehensive statistical validation of repeatability across a larger sample size remains an important question for future exploration.

### 4.2. Relationship Between the Crack Length and Transmission Coefficient

Considering both accuracy and practical applicability, the association of crack length with the envelope amplitude is described using a linear model. According to the geometric relationship, the projected crack length at position 1 is equal to the actual fatigue crack length. Furthermore, as discussed in Section 3.6, when the projected crack length in a C(T) specimen exceeds 10.39 mm, it can be effectively detected by the wireless ultrasonic sensor. A comparison of the experimental data reveals that the crack lengths during steps 3B, 4B, and 5B meet the detection requirements, and the ultrasonic test results are therefore valid. Based on the analysis in Section 3.4, when the tensile load is less than 6.67 kN, the crack faces come into contact (i.e., the crack closes), making the attenuation of the ultrasonic transmission signal less sensitive to changes in crack length and therefore unsuitable for quantitative assessment. Based on the above considerations, this section establishes a valid correspondence between the fatigue crack length of the C(T) specimen during steps 3B, 4B, and 5B and the normalized envelope amplitude of the ultrasonic signal collected at position 1 under tensile loads of 6.67 kN, 10.01 kN, and 13.34 kN, respectively, near the waveform at 12 μs.

To construct a standardized envelope amplitude–crack length linear relationship model, three schemes were considered, yielding seven sets of standardized envelope amplitude values corresponding to the crack length of the C(T) specimen: (1) tensile load of 6.67 kN; (2) tensile load of 10.01 kN; (3) tensile load of 13.34 kN; (4) mean values of (1) and (2); (5) mean values of (2) and (3); (6) mean values of (1) and (3); and (7) mean values of (1), (2), and (3). As an example, Figure 11 presents the model fitted to the first dataset using the least squares method.

**Figure 14:**
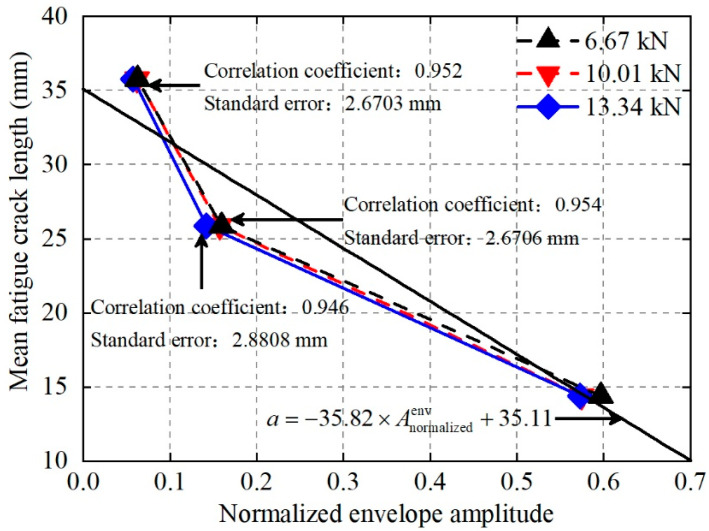
Curves of the test and fitted average crack lengths versus the transmission coefficients.

As shown in Figure 14, the root mean square error (RMSE) serves as a metric for assessing the fit quality of a model. The RMSE values corresponding to tensile loads of 6.67 kN, 10.01 kN, and 13.34 kN are 2.6703 mm, 2.6706 mm, and 2.8808 mm, respectively. The RMSE values calculated from the other datasets are presented in Figure 11. No overarching trend in the RMSE can be discerned, given that the datasets for different tensile loads were acquired separately. Therefore, equal weights should be assigned to the RMSE values obtained in all the scenarios. Accordingly, the average RMSE is employed as a representative parameter for general assessment. The average RMSEs for the various scenarios were computed and are presented in Table 3. Among these, the seventh scenario yields the smallest average RMSE. Therefore, the chosen fitting expression serves to characterize the *a*-*T* correlation for the C(T) specimen.

### 4.3. Model for the Envelope Amplitude and Fatigue Loading Cycles

By combining Equations (13) and (14), the *a*-*N* relationship associated with the modified secant method can be written as follows:

(13) $$\left\{\begin{array}{l} N=N_{3A}+\left(7.26\times 10^{7}\right)\times \int_{a_{3A}}^{a_{c}}F_{3}{\left(a\right)}^{2.84}da,a_{3a}\leq a\leq a_{5a}\\ N=N_{3A}+\left(7.26\times 10^{7}\right)\times \int_{a_{3a}}^{a_{5a}}F_{3}{\left(a\right)}^{2.84}da+\left(2.30\times 10^{7}\right)\times {\int_{a_{5a}}^{a_{c}}F_{3}\left(a\right)}^{2.84}da,a\geq a_{5a} \end{array}\right.$$

F_3_(*a*) is defined as follows: $F_{3}\left(a\right)=\frac{{\left(1-9.84a\right)}^{3}}{1.77+1.00\times 10^{2}a-2.13\times 10^{3}a^{2}+1.54\times 10^{4}a^{3}+3.30\times 10^{4}a^{4}-5.17\times 10^{5}a^{5}}$.

Based on Equation (11), substitution of the *a*-*T* relationship derived in Section 4.1 provides the following *N*-*T* expression:

(14) $$\left\{\begin{array}{l} N=N_{3A}+\left(-2.68\times 10^{6}\right)\times \int_{T_{3B}}^{T_{c}}F_{4}{\left(a\right)}^{2.84}dT,T>T_{5B}\\ N=N_{3A}+\left(-2.68\times 10^{6}\right)\times \int_{T_{3B}}^{T_{5B}}F_{4}{\left(a\right)}^{2.84}dT+\left(-8.48\times 10^{5}\right)\times \int_{T_{5B}}^{T_{c}}F_{4}{\left(a\right)}^{2.84}\;dT,T\leq T_{5B} \end{array}\right.$$

where $F_{4}\left(A\right)=\frac{{\left(0.65+0.36A\right)}^{3}}{3.35-0.34A-0.66A^{2}-0.68A^{3}-0.11A^{4}+0.04A^{5}}$. Similarly, the relationship for the five-point incremental polynomial method is given by the following:

(15) $$\left\{\begin{array}{l} N=N_{3A}+\left(-3.51\times 10^{5}\right)\times \int_{T_{3B}}^{T_{c}}F_{2}{\left(a\right)}^{1.52}dT,T>T_{5B}\\ N=N_{3A}+\left(-3.51\times 10^{5}\right)\times \int_{T_{3B}}^{T_{5B}}F_{2}{\left(a\right)}^{1.52}dT+\left(-1.90\times 10^{5}\right)\times \int_{T_{5B}}^{T_{c}}F_{2}{\left(a\right)}^{1.52}\;dT,T\leq T_{5B} \end{array}\right.$$

Two experiments were conducted under tensile loads of 6.67 kN, 10.01 kN, and 13.34 kN, and the original transfer coefficients derived from ultrasonic measurements in experiments 3B, 4B, and 5B were verified. In addition, in the case of the final fracture of the C(T) specimen, the transfer coefficient was set to zero. Numerical integration was executed utilizing the Gauss–Kronrod integration scheme. Using Equations (14) and (15), the *T*-*N* curves were computed and subsequently compared with the experimental measurements, as shown in the analysis results in Table 4.

As shown in Table 4, all correlation coefficients between the calculated results and experimental measurements are greater than 0.99. In Experiments 1 and 2, the errors between the predictions obtained using the modified secant method under different tensile loads and the experimental results are consistently approximately 8% and 3%, respectively. The deviations between the predictions obtained using the five-point incremental polynomial method and the experimental results are consistently approximately 13%. These findings indicate that the input parameters obtained from different experiments can influence the reliability of the predicted fatigue life, whereas the impact of the input parameters from the same set of experiments under different tensile loads on the fatigue life prediction model is relatively small. For different methods, the standard errors significantly differ, indicating that the choice of method has a substantial influence on the results. This research indicates that the model derived from the modified secant method more closely corresponds with the experimental measurements. Therefore, the envelope amplitude obtained from transmitted ultrasonic signals under higher tensile loads offers a more accurate representation of the actual crack state in C(T) specimens. It should be noted that the work presented in this paper is preliminary. This work serves as a foundational proof-of-concept to validate the feasibility and field application potential of a wireless sensing device. Although the empirical model proposed in this paper exhibits strong repeatability under controlled conditions, subsequent research is necessary to expand its application by considering critical variables, including specimen size, loading profiles, and material types. Additionally, given high sensitivity to fatigue damage, the characteristics (e.g., phase shifts, time-of-flight variations, and acoustic nonlinearity) require further study in order to improve fatigue life prediction via wireless sensing methods.

## 5. Conclusions

(1) The experimentally measured fatigue crack data were validated in accordance with the ASTM standard. Different stages in the fatigue crack propagation process were identified through morphological analysis, and the reasons for the significant characteristics observed in each stage were discussed.
(2) Waveform-based analysis revealed a strong consistency between the crack opening and closing states and the corresponding amplitude variations in the waveform. Furthermore, the relationship between crack length and the transmission coefficient of ultrasonic signals collected via the wireless system was determined.
(3) The modified secant method together with the five-point incremental polynomial method were used to establish the *da*/*dN*-Δ*K* relationship for the compact-tension specimen. On this basis, and in accordance with Paris’s law and Miner’s rule, the association of crack length with loading cycles was formulated.
(4) A fatigue crack propagation model for the C(T) specimen was constructed utilizing signals from a wireless ultrasonic sensing system through two distinct approaches. Compared with the experimental results, the model derived from the five-point incremental polynomial method exhibited good consistency in terms of curve trends, and the modified secant method model showed an even closer correspondence with the experimental results.

## Figures and Tables

**Table 1:** Detailed loading parameters for the C(T) specimen fatigue test.

Step Number	Loading Type	Description	Purpose
0B	Multistage static	From 0.44 kN to 13.34 kN, loading in 5 steps. The second level of load is 3.34 kN, and each subsequent stage of load increases compared with the previous level of 3.34 kN.	Benchmark ultrasonic measurement
1A	Cyclic loading	5 Hz sinusoidal load with a loading range from 0.44 kN to 13.34 kN; 15,000 cycles.	Crack prefabrication
1B	Multistage static	From 0.44 kN to 13.34 kN, loading in 5 steps. The second level of load is 3.34 kN, and each subsequent stage of load increases compared with the previous level of 3.34 kN.	Ultrasonic measurement
2A	Cyclic loading	5 Hz sinusoidal load with a loading range from 0.44 kN to 13.34 kN; 10,000 cycles.	Crack propagation
2B	Multistage static	Same as 1B	Ultrasonic measurement
3A	Cyclic loading	Same as 2A	Crack propagation
3B	Multistage static	Same as 2B	Ultrasonic measurement

**Table 2:** Paris’s model parameters under different crack growth rate fitting methods.

Test Number	Fitting Method	Before Correction			After Correction		
		*C*	*m*	Correlation Coefficient with Growth Rate	*C*_eff_	*m*_eff_	Correlation Coefficient with Growth Rate
1	Modified secant method	−47.24	2.1127	0.9769	−24.67	2.8419	0.9786
	Five-point incremental polynomial method	−33.86	1.2719	0.9999	−15.98	1.5215	0.9999
2	Modified secant method	−51.52	0.9340	0.9634	−25.25	2.9431	0.9984
	Five-point incremental polynomial method	−37.46	0.0357	0.9999	−18.83	1.9671	0.9999

**Table 3:** Establishment and comparison of linear relationships under different schemes and parameter results.

Scenario Index	Fitting Parameters in *a* = *a*_1_*T* + *a*_2_		Individual RMSE Calculated from the Test Data for Different Tensile Loads (mm)			Average RMSE (mm)
	*a*_1_	*a*_2_	Tensile Load at 6.67 kN	Tensile Load at 10.01 kN	Tensile Load at 13.34 kN	
1	−35.83	35.22	2.6704	2.6706	2.8808	2.7406
2	−37.48	35.26	2.7149	2.629	2.8409	2.7283
3	−36.66	34.78	2.7356	2.6485	2.8221	2.7354
4	−36.63	35.18	2.6812	2.6399	2.852	2.7244
5	−37.07	35.02	2.7208	2.634	2.8267	2.7272
6	−36.24	34.95	2.6866	2.6434	2.8372	2.7224
7	−36.91	35.21	2.6867	2.6309	2.8429	2.7202

**Table 4:** Comparison of prediction effect parameters for different input data.

Test Number	Tensile Load Level (kN)	Fitting Method	Predict Fatigue Life (Cycles)	Standard Error (Cycles)	Correlation Coefficient
1	6.67	Modified secant method	45,907	3447	0.994
		Five-point incremental polynomial method	43,303	5367	0.998
	10.01	Modified secant method	45,908	3444	0.994
		Five-point incremental polynomial method	43,304	5361	0.998
	13.34	Modified secant method	45,916	3420	0.993
		Five-point incremental polynomial method	43,324	5303	0.997
2	6.67	Modified secant method	52,444	2273	0.994
		Five-point incremental polynomial method	46,948	5669	0.999
	10.01	Modified secant method	52,452	2264	0.994
		Five-point incremental polynomial method	46,966	5635	0.999
	13.34	Modified secant method	52,452	2258	0.994
		Five-point incremental polynomial method	46,967	5636	0.999

## Data Availability

The data that support the findings of this study are available from the corresponding author upon request.

## Abbreviations

The following abbreviations are used in this manuscript:

C(T) specimen	compact tension specimen
SIF	stress intensity factor
NDT	nondestructive testing
ASTM	American Society for Testing and Materials
CMOD	crack mouth opening displacement
